# Items

**Published:** 1880-11

**Authors:** 


### Items.

The sixth annual meeting of the Indiana, Illinois and Kentucky Tri-State Medical Society will be held at Masonic Temple, Louisville, Ky., on Tuesday, Wednesday, Thursday and Friday, 9th, 10th, 11th and 12th November, 1880. Convenes Tuesday, 9th, at 9 a. m.

*Order of Business.*—1. Prayer, by the Rev. Jas. P. Boyce; 2. Welcome address, by Mayor J. G. Baxter; 3. Welcome address in behalf of Kentucky, by Gov. Blackburn; 4. Welcome address in behalf of the profession, by Dr. E. R. Palmer; 5. Response. 2 p. m.—6. Report of Committee on Arrangements, by Dr. W. W. Senteny, Louisville; 7. Report of Secretary and the Committee on Publication, by Dr. G. W. Burton, Mitchell, Ind.; 8. Report of Treasurer, by Dr. F. W. Beard, Vincennes, Ind.; 9. Call for volunteer papers; 10. New method of closing amputation stumps by "mattrass sutures," by Dr. A. C. Bernays, St. Louis; 11. Typho-malarial fever, or mongrel hybrid compound fevers, by Dr. Chas. F. Reber, Shelbyville, Ind.; 12. Something in connection with State medicine, by Dr. Thad. M. Stephens, Indianapolis. 7:30 p. m.—President's address, by Dr. H. B. Buck; Microscopic exhibitions, by Dr. J. B. Marvin, of Louisville, and assistants.

*Second Day.*—Chairman of Sections: Surgery—Dr. J. R. Weist, Richmond, Ind.; Practice of Medicine—Dr. B. M. Griffith, Springfield, Ill.; Obstetrics—Dr. Geo. B. Walker, Evansville, Ind.; Gynæcology—Dr. Ed. W. Jenks, Chicago; Pathology and Microscopy—Dr. J. Gardner, Bedford, Ind.; Ophthalmology—Dr. J. E. Harper, Evansville, Indiana; State Medicine—Dr. Horace Wardner, Anna, Illinois.

*Regular papers.*—1. Tubercular cerebral meningitis, by Dr. J. B. Richardson, Louisville; 2. The early manifestations of insanity, by Dr. B. W. Stone, Hopkinsville, Ky.; 3. Insanity, home and asylum treatment compared, by Dr. C. C. Forbes, Louisville; 4. Mediæval medicine, by Dr. H. C. Fairbrother, East St. Louis; 5. Hæmiopia, mechanism of, causation of, the theory of total decussation of fibers of the optic tracts at the chiasma, by Dr. Wm. Dickinson, St. Louis; 6. Embliopia from disuse, by Dr. Williams, of Cincinnati; 7. Architectural hygiene, by Dr. E. W. Gray, Bloomington, Ill.; 8. Treatment of ununited fractures, by Dr. Duncan Eve, Nashville; 9. The histopathology of scarlet fever, by Dr. J. V. Block, Jacksonville, Ill.; 10. Chemistry versus vitality, by Dr. John T. Freeland, Freelandsville, Ind.; 11. Effects of the sulphate of quinia during pregnancy and after delivery, by Dr. F. G. Ford, Russellville, Ind.; 12. Investigation of utero-placental circulation, by Dr. Ferdinand Smith, Frankfort, Missouri; 13. The use of atropia sulphate in the collapse of cholera, cholera morbus, and kindred affections, by Dr. S. P. Craik, Stanford, Ky.; 14. What is the matter with my patients? by Dr. A. W. Spain, Poseyville, Ind.; 15. The successful physician, by Dr. W. A. Hunt, Lynnville, Ind.; 16. So called changes in types of disease, by Dr. B. R. Helm, Carlisle, Ind.; 17. Dressing of wounds, by Dr. David Prince, Jacksonville, Ill.; 18. Remarks on the pathology and treatment of epilepsy, by Dr. J. S. Jewell, Chicago; 19. Mind in its relation to the healing art, by Dr. C. H. Spillman, Harrodsburg, Ky.; 20. Thoughts on progressive medicine, by Dr. J. W. Singleton, Paducah, Ky.; 21. Surgical treatment of dysmenorrhœa, by Dr. J. P. Thomas, Pembroke, Ky.; 22. Mind as a therapeutic agent, by Dr. J. J. Speed, Louisville; 23. Early management of infancy, by Dr. J. A. Larrabee, Louisville; 24. Report of cases, by Dr. J. W. Thompson, Paducah, Ky.; 25. Puerperal convulsions, by Dr. W. W. Cleaver, Lebanon, Ky.; 26. Neuralgia of infra- and supra-orbital nerves, by Dr. Thos. F. Rumbold, St. Louis; 27. Arrest of evolution versus maternal impressions, by Dr. Arch. Dickson, Henderson, Ky.; 28. Aid of microscope in diagnosing disease, by Dr. W. S. Ross, Evansville, Ind.; 29. Pernicious fever, by Dr. C. H. Norred,

Lincoln, Ind.; 30. Conjectures on tactile sensibility, by Dr. W. J. Cole, Blairsville, Ind.; 31. A few cases of rupture of the lungs, by Dr. J. P. Ross, Chicago; 32. Total extirpation of the uterus, by Dr. E. C. Dudley, Chicago, Ill.; 33. Some unusual manifestations of hysteria, by Dr. Edwin Walker, Evansville, Ind.; 34. Cox's operation for impassable stricture, by Dr. A. N. Johnson, Danville, Ky.; 35. Therapeutic value of some of our indigenous plants, by Dr. Lucian McDowell, Flemingsburg, Ky.; 36. Use of forceps in obstetrical practice, by Dr. S. B. Friror, Catlettsburg, Ky.; 37. Wound dressing, by J. H. Letcher, Henderson, Ky.; 38. Contagiousness of phthisis, by Dr. S. M. Letcher, Richmond, Ky.; 39. Ophthalmic therapeutics, by Dr. John Green, St. Louis; 40. The proper management of natural labor, by Dr. A. D. Price, Harrodsburg, Ky.; 41. Questions in lithotrity, by Dr. R. A. Vance, Cincinnati, O.; 42. Typho-malarial fever, by Dr. J. W. Compton, Evansville, Ind.; 43. The dangers in our present educational system, by Dr. W. W. Blair, Princeton, Ind.; 44. Ovarian tumors, by Dr. Ed. Borck, St. Louis; 45. Pyelitis and its relation to Bright's disease, by Dr. I. N. Danforth, Chicago; 46. Diseases of the eye in connection with general diseases, by Dr. W. W. Cheatham, Louisville, Ky.; 47. Post partum injection of uterine cavity, by Dr. B. J. Day, Evansville, Ind.; 48. Obstetrics, by Dr. Walter Coles, St. Louis; 49. A physician's problems in psychiatry, by Dr. C. H. Hughes, St. Louis; 50, ———, Dr. Ohman Damisnell, St. Louis; 51. Prevention and treatment of mammary abscess, by Dr. W. H. Taylor, Cincinnati, O.; 52. Treatment of diphtheria by quinine inhalations, by Dr. Gustave Zinke, Cincinnati, O.; 53. Non-astringent caustic treatment of conjunctival inflammations, by Dr. W. W. Seely, Cincinnati, O.; 54. Dyspepsia, by Dr. H. Illoway, Cincinnati, O.; 55. Extirpation of parotid gland, by Dr. J. P. Harvey, Grove City, Ill.; 56. ———, W. D. Davis, Cincinnati, O.; 57. Public address, by Dr. Theophilus Parvin, Indianapolis; 58. Porro's method in the Cæsarean operation, by Dr. J. W. McCormick, Bowling Green; 59. Lactation, by Dr. Theophilus Parvin, Indianapolis; 60. Objective points in the treatment of phthisis, by Dr. Wm. Porter, St. Louis; 61. Modus operandi of cod liver oil, by Dr. Samuel Nickels,

Cincinnati, O.; 62. Treatment of stricture of bulb of the urethra, by Dr. W. H. Ford, St. Louis; 63. Management of children under surgical treatment, by Dr. C. A. Le Boutellier, Cincinnati, O.; 64. Recent investigations in pathology, by Dr. G. H. Hallister, Cincinnati, O.; 65. Color blindness, by Dr. M. F. Coomes, Louisville; 66. "Dr. Tanner," by Dr. J. Hays, Louisville; 67. "Risks of surgical operations," by Dr. J. Hale, Owensboro, Ky. If desirable, the different sections will hold separate meetings in the afternoons.

*Officers for* 1880.—President, Dr. H. B. Buck, Springfield, Ill.; First Vice President, Dr. S. E. Monford, Princeton, Ind.; Second Vice President, Dr. J. P. Thomas, Pembroke, Ky.; Third Vice President, Dr. T. J. Ford, Russelville, Ill.; Secretary, Dr. G. W. Burton, Mitchell, Ind.; Treasurer, Dr. F. W. Beard, Vincennes, Ind.; Chairman Committee of Arrangements, Dr. W. W. Senteny, Louisville; Chairman Committee on Programme, Dr. W. W. Cheatham, Louisville; Committee on Transportation, Drs. R. O. Cowling, Geo. W. Griffths, and John A. Brady, Louisville; Chairman Committee on Entertainment, Dr. T. P. Satterwhite.

*Railroad Rates.*—L. & N. and tributaries, three cents a mile each way. Short Line and tributaries, one and one-third fare round trip. L., N. A. & Chicago, one-half fare round trip. Jeffersonville, Madison & Indianapolis, two and one-half cents a mile each way. Ohio & Mississippi, one and one-third fare.

*Hotel Rates.*—Louisville Hotel, $2.50 per day; Galt House, $2.50 per day; Willard Hotel, $2.00 per day; Alexander's Hotel, $2.00 per day; Fifth Avenue Hotel, $2.00 per day; St. Cloud Hotel, $1.50 per day.

MALARIA IN NEW ENGLAND.—The increase of malarial diseases, especially intermittent fever, in New England, is exciting considerable attention (*Med. and Surg. Reporter*) The Massachusetts State Board of Health, with its accustomed zeal and energy, has taken hold of the subject, and has already collected a mass of facts which is astounding, and confirms to the fullest extent the rumors heretofore prevalent. The form of ague near near Providence, R. I., is said almost to equal the worst forms in the Southern States.

